# Real-Time In Vivo Imaging of Mouse Left Ventricle Reveals Fluctuating Movements of the Intercalated Discs

**DOI:** 10.3390/nano10030532

**Published:** 2020-03-16

**Authors:** Fuyu Kobirumaki-Shimozawa, Tomohiro Nakanishi, Togo Shimozawa, Takako Terui, Kotaro Oyama, Jia Li, William E. Louch, Shin’ichi Ishiwata, Norio Fukuda

**Affiliations:** 1 Department of Cell Physiology, The Jikei University School of Medicine, Tokyo 105-8461, Japan; n.tommy05@jikei.ac.jp (T.N.); oyama.kotaro@qst.go.jp (K.O.); 2 Technical Division, School of Science, The University of Tokyo, Tokyo 113-0033, Japan; shimozawa@phys.s.u-tokyo.ac.jp; 3 Department of Anesthesiology, The Jikei University School of Medicine, Tokyo 105-8461, Japan; pu-cha@jikei.ac.jp; 4 Quantum Beam Science Research Directorate, National Institutes for Quantum and Radiological Science and Technology, Gunma 370-1292, Japan; 5 Institute for Experimental Medical Research, Oslo University Hospital and University of Oslo, 0450 Oslo, Norway; jia.li@medisin.uio.no (J.L.); w.e.louch@medisin.uio.no (W.E.L.); 6 KG Jebsen Center for Cardiac Research, University of Oslo, Oslo, Norway; 7 Department of Physics, Faculty of Science and Engineering, Waseda University, Tokyo 169-8555, Japan; Ishiwata@waseda.jp

**Keywords:** myocardium, gap junction, action potential, heart

## Abstract

Myocardial contraction is initiated by action potential propagation through the conduction system of the heart. It has been thought that connexin 43 in the gap junctions (GJ) within the intercalated disc (ID) provides direct electric connectivity between cardiomyocytes (electronic conduction). However, recent studies challenge this view by providing evidence that the mechanosensitive cardiac sodium channels Na_v_1.5 localized in perinexii at the GJ edge play an important role in spreading action potentials between neighboring cells (ephaptic conduction). In the present study, we performed real-time confocal imaging of the CellMask-stained ID in the living mouse heart in vivo. We found that the ID structure was not rigid. Instead, we observed marked flexing of the ID during propagation of contraction from cell to cell. The variation in ID length was between ~30 and ~42 μm (i.e., magnitude of change, ~30%). In contrast, tracking of *α*-actinin-AcGFP revealed a comparatively small change in the lateral dimension of the transitional junction near the ID (i.e., magnitude of change, ~20%). The present findings suggest that, when the heart is at work, mechanostress across the perinexii may activate Na_v_1.5 by promoting ephaptic conduction in coordination with electronic conduction, and, thereby, efficiently transmitting excitation-contraction coupling between cardiomyocytes.

## 1. Introduction

Contraction of mammalian cardiomyocytes is regulated via excitation-contraction coupling on a graded basis. When the cellular membrane is depolarized via entry of Na^+^ into the myocyte through Na^+^ voltage channels (Na_v_1.5), Ca^2+^ enters the myocyte via sarcolemmal *L*-type Ca^2+^ channels localized in the T-tubules (see References [1,2] and references therein). This Ca^2+^ does not directly activate myofilaments but triggers the release of Ca^2+^ from the sarcoplasmic reticulum (SR) via the Ca^2+^-induced Ca^2+^ release mechanism, which results in the binding of Ca^2+^ to troponin C and the ensuing attachment of myosin to thin filaments. Because the T-tubules run parallel and adjacent to the Z discs in cardiomyocytes, the intracellular Ca^2+^ concentration ([Ca^2+^]*_i_*) does not increase homogenously in the myocyte. Rather, [Ca^2+^]*_i_* is the highest proximal to the T-tubules and relatively low in the distal regions such as the thick-thin filament overlap region. Accordingly, cardiac myofilaments are only partially activated even at the peak of systole (see References [1,2] and references therein). [Ca^2+^]*_i_* starts to fall prior to myocardial relaxation. Lowering [Ca^2+^]*_i_* dissociates Ca^2+^ from troponin C, and results in dissociation of myosin from thin filaments, i.e., relaxation. 

**Figure 1:**
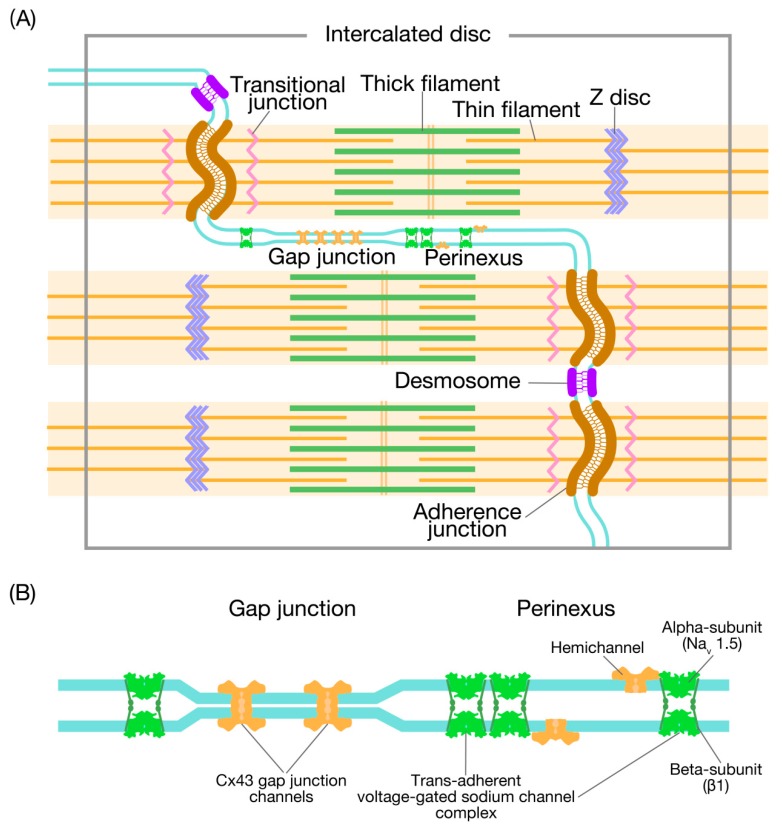
Illustration showing the domains associated with the intercalated disc. (**A**) The intercalated disc (ID) between cells predominantly accommodates three types of intercellular junctions: the adherens junction, the desmosome, and the gap junction (GJ). Thin filaments from the last sarcomere of the myofibrils insert in the adherens junction passing through a transitional junction (TrJ), which is rich in Z disc proteins (hence, an immature Z disc). At the edge of the GJ, there is an extracellular nanodomain, i.e., the perinexus (see (**B**) for details). See [6]. (**B**) Illustration showing the GJ and adjacent perinexus. The GJ comprises a cluster of Cx43 channels, docking within the narrow 2-nm gap. While the *α*-domain of Na_v_1.5 has a relatively scarce presence in the GJ, the sodium channels are enriched in the perinexus. In this case, the non-pore-forming *β*-subunit of Na_v_1.5 cross-links the two sides of the perinexus. Undocked Cx43 hemichannels are also expressed in the perinexus. See [8].

It is well established that cardiomyocytes interact with each other at their ends through the specialized membrane complex known as the intercalated disc (ID). The ID has a plicate structure, which results in a folded appearance when examined in longitudinal sections and the appearance of cellular membrane loops when viewed transversely as loops (see References [3,4] and references therein). The width of the folds varies considerably in different areas of the heart, from ~500 nm to ~2 μm [5]. This variation is dependent on the presence of the gap junction (GJ), which provides a pathway for cell-to-cell electrical conductance with low resistance. There are three major types of cell-cell junctions within the ID: (1) the GJ where the electrical signal is transmitted from cell to cell, (2) the adherens junction where mechanical force is transmitted from cell to cell, and (3) the desmosome that binds cells together and anchors the cytoskeletal desmin filaments (Figure 1A) (see References [6,7] and references therein). In addition, there are regions in the ID, composed of caveolae, coated pits, and the spectrin-associated signaling domains with no intercellular junctions and no clear function. 

There is a general consensus that the GJ provides direct electrotonic conduction between connected myocytes, which enables conduction of the action potential and triggers the heartbeat. It has been considered, for more than a half century, that connexin 43 (Cx43), which is highly expressed in the GJ, which provides this electric connectivity (electronic conduction). However, a set of recent studies challenges this view, since GJ channel aggregates are very rarely seen in birds, reptiles, amphibians, and fish species, and are regularly found only in mammals [8,9,10,11]. If Cx43 is, in fact, essential for cell-to-cell communication of depolarization in the heart, how then can electrical conduction be accomplished across the hearts of these animals (as discussed in Reference [8])? It seems rather that, although Cx43-based electronic conduction operates at least in the heart of mammals, an alternative mechanism is likely to co-operate in promoting the cell-to-cell electrical depolarization.

In myocardium of virtually all animal species, there are regions within the ID around the GJ where the membranes of contacting cells separate, but remain in close proximity by ~100 nm [8,12]. These peri-junctional regions are known as perinexii, and are located at the edge of the Cx43-containing GJ (see Figure 1B, [12]). Recent studies demonstrated that the cardiac sodium protein Na_v_1.5 (*α*-subunit) is highly expressed in these perinexii in mammalian ventricular and atrial muscles [13,14], and that non-pore-forming *β* subunits are structurally essential in maintaining the close membrane apposition [15,16]. The induction of an action potential via Na_v_1.5 from the extracellular space is termed as “ephaptic conduction” [12] and requires a distance between the membranes of connecting cells, which is <30 nm (i.e., the Mori limit, see Reference [8]). The widely accepted mechanism of ephaptic conduction is as follows: First, when two myocytes are connected, depolarization of one of the cells via Na_v_1.5 causes withdrawal of Na^+^ from the restricted extracellular cleft in the perinexii. The resulting depletion of positive charge from the perinexus lowers the local extracellular potential, which results in a relative decrease in the transmembrane potential of the contacting cell (i.e., intracellular potential minus extracellular potential). Consequently, Na_v_1.5 in the contacting cell is activated, and triggers excitation-contraction coupling. Although the role of electric conduction has been challenged in recent reports [17,18,19], we consider that the two types of action potential conduction, i.e., ephaptic conduction and electric conduction, likely cooperatively spread depolarization across the myocardium.

It should likewise be noted that Na_v_1.5 plays a variety of functional roles in cardiac physiology (e.g., Reference [20]) in the pathophysiology of arrhythmias (e.g., Reference [21]), ischemic heart disease (e.g., Reference [22]), and heart failure (e.g., Reference [23]). It has been reported that mutations in Na_V_1.5 and in the Na_V_1.5-interacting genes that modify its function and trafficking cause arrhythmic syndromes and cardiomyopathies with contractile dysfunction (see References [23] and references therein).

To the best of our knowledge, no studies have investigated whether or not structural changes and, accordingly, functional alterations occur within the ID. In this regard, insight is particularly lacking into the respective roles of the GJ and perinexii in the in vivo beating heart. By employing in vivo nano-imaging, we presently investigated whether the ID structure is altered during propagation of contraction from cell to cell in the living mouse heart. We found that the structure of the plasma membrane regions within the ID were dramatically variable and show fluctuating movements in vivo. The physiological implications of these findings are discussed.

## 2. Materials and Methods 

This study was performed in accordance with the Guidelines on Animal Experimentation of The Jikei University School of Medicine. The study protocol was approved by the Animal Care Committee of The Jikei University School of Medicine and the Recombinant Gene Research Safety Committee of The Jikei University School of Medicine. All experiments were performed at The Jikei University School of Medicine.

### 2.1. CellMask Treatment in the Heart of Living Mice In Vivo

Myocardial membranes, including T-tubules, were stained by CellMaskTM Orange (CellMask, Life Technologies Inc., Tokyo, Japan) according to the method used in our previous study [24,25]. CellMask was dissolved in PBS (-) (termed “CellMask solution”), and administered to anesthetized open-chest, temperature-regulated mice (see below) via a small piece of gauze placed on the left ventricular (LV) surface. The 0.1 μg/mL CellMask solution was gently dropped onto the gauze for 5 min using a pipette. 

### 2.2. α-Actinin-AcGFP Expression in the Heart of Living Mice In Vivo 

The adenovirus vector (ADV) was constructed based on our previous studies [25,26]. Recombinant adenoviruses encoding mouse *α*-actinin-3-AcGFP (Genbank/EMBL/DDBJ accession no. NM_013456) (Ad-actinin-AcGFP) were constructed using the AdMax adenovirus vector creation kit (Microbix Biosystems Inc., Toronto, ON, Canada).

The ADV was then purified using the Vivapure AdenoPACK 20 purification kit (Sartorius AG, Weender Landstr, Göettingen, Germany) [26], and concentrated by ultrafiltration to yield 1 × 10^11^–1 × 10^12^ viral particles per mL in PBS (-). ADVs were stored at −80 °C for up to two months. 

ADV was injected into the heart of male BALB/c mice (aged 3–8 weeks) anesthetized with ~2% isoflurane as in our previous study [26]. In brief, left thoracotomy was performed in order to visualize the anterior surface of the LV under ventilation. The animal body temperature was maintained at 38 °C. PBS (-) containing ADV at various viral titers was injected into the epicardial surface of the central region of the LV (10 µL in ~3 × 3 mm^2^, ~10 spots) using a 1 mL syringe pump with a 32G needle. Two days after chest closure, the mouse was anesthetized again with ~2% isoflurane and ventilated, and the anterior thoracic wall was removed by cutting the ribs, muscles, and intercostal arteries with an electric scalpel for in vivo cardiac sarcomere imaging.

### 2.3. Microscopic System

The details of the microscopic system for in vivo nano-imaging have been described in our previous studies [25,26,27]. In brief, an upright microscope (BX-51WI, Olympus Co., Tokyo, Japan) combined with a Nipkow confocal scanner (CSU21, Yokogawa Electric Co., Tokyo, Japan) and an electron multiplying CCD camera (iXonEM+, Andor Technology Ltd., Belfast, Northern Ireland) were used at a 512 × 512 (or 512 × 170) pixel resolution at an exposure time of 28 (or 9.8) ms. A 60× water immersion lens (LUMPLFLN 60 × W, N/A 1.00, Olympus Co., Tokyo, Japan) was used to visualize the LV surface. 

CellMask-treated myocytes were excited at 532 nm (MiniGreen FCIM-100, Snake Creek Lasers, Friendsville, PA, USA), and the resultant fluorescence was detected passing a BA575IF emission filter (Olympus Co., Tokyo, Japan). AcGFP-expressing myocytes were excited by a 488-nm laser light (HPU50211-PFS, Furukawa Electric Co., Tokyo, Japan), and the resultant fluorescence signals using a BA510–550 bandpass emission filter (Olympus Co., Tokyo, Japan) were detected.

Our optics system with the Nipkow confocal unit enabled imaging at the previously mentioned spatial and temporal resolutions at a depth of up to ~150 μm in the mouse heart in vivo in the case of either CellMask or *α*-actinin-AcGFP.

### 2.4. In Vivo Nano-Imaging

Nano-imaging was performed in accordance with our previous studies [25,26,27]. The anesthetized and ventilated open-chest mouse was placed on a custom-made microscope stage (250 × 350 mm), and the animal was warmed to 38 °C throughout imaging. 

A coverslip (0.04–0.06 mm thickness, 12 mm diameter, No.000, Matsunami Glass Ind., Osaka, Japan) was gently attached to the LV surface at two points (~2 mm apart) using glue (Aron Alpha, TOA GOSEI Co., Tokyo, Japan). The position of the coverslip was carefully controlled using a custom-made micro-manipulator (SIGMAKOKI Co., Tokyo, Japan). In the present study, for both CellMask and AcGFP, imaging was performed at a low heart rate under deep anesthesia with ~5% isoflurane. Heart arrest was defined as no signal of electrocardiogram (with >~5% isoflurane). The ventilator was turned off during imaging.

## 3. Analyses

All data were analyzed using ImageJ software (National Institute of Health, Bethesda, MD, USA). In the experiments with CellMask, cells were imaged within the first or second layer of the LV epicardium. The ID was located between two cells, labelled Cell 1 (at left) and Cell 2 (at right), by determining the position with peak fluorescence intensity (F.I.). The ID was then traced using the “Segmented lines” function. The length of the ID was analyzed using the “Measure” function, with the start and end points defined as the points of intersection between the longitudinal and lateral membrane. The cell width was defined as the length of the straight line between these two points. To quantify the relative displacement of the ID, the “deviation distance” was defined as the distance from the midpoint of the straight line to the nearest measurable central region of the ID. The deviation distance values were defined as negative and positive, respectively, by corresponding to displacement of the ID to the left and right side of the cell width line. 

As in the CellMark experiments, *α*-actinin-AcGFP was tracked in cells located in the first or second layer of the surface of the LV. The positions exhibiting peak F.I. along the TrJ or Z disc were identified, and cell width measurements defined as described above for measurements of the ID. The “deviation distance” was similarly defined as the distance between the vertical dimension and the central region of the TrJ. The average length of 10 sarcomeres next to the measured TrJ was defined as average sarcomere length (SL) (see Reference [26] for details of SL measurement). As above, the cells located on the left and right side of the measured TrJ were termed Cell 1 and Cell 2, respectively. 

## 4. Results and Discussion

**Figure 2:**
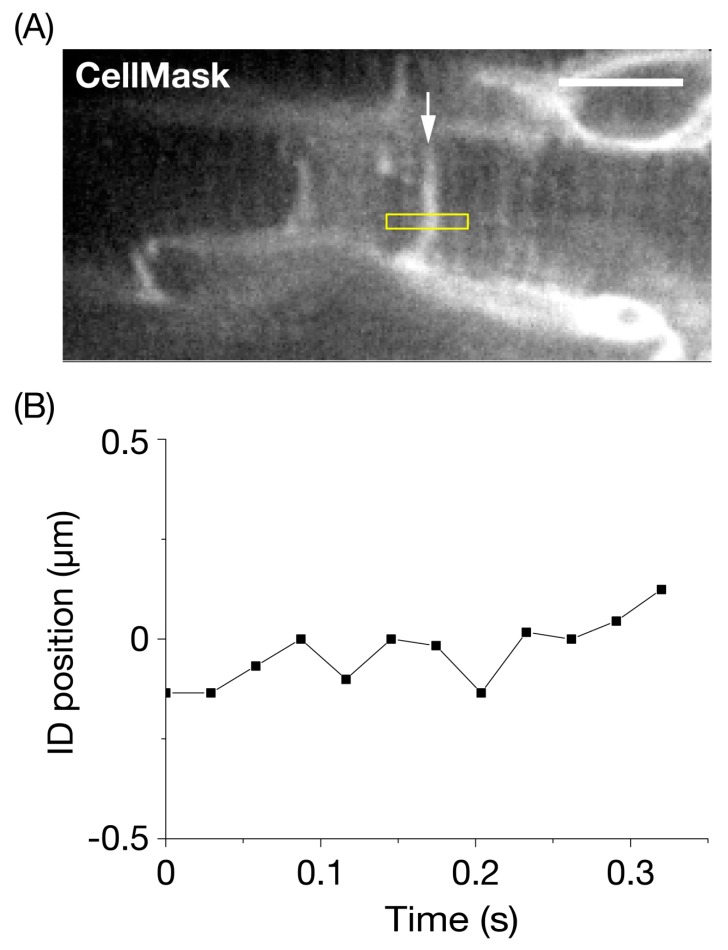
Precision analysis for the longitudinal position of the intercalated disc in the left ventricle of the mouse heart at rest in vivo. (**A**) Confocal image of connecting myocytes. The intercalated disc (ID) is shown by an arrow with a higher fluorescence signal than in *T*-tubules (cf. [24,25]). The membrane systems in the surface of the left ventricle were stained by CellMask, according to our previously reported procedure (see Materials and Methods). The longitudinal fluctuation of an ID section (width, 5 pixels (=1.05 μm)) in the yellow rectangle was analyzed under the arrested condition via deep anesthesia with >~5% isoflurane. Bar on the top right indicates 10 μm. (**B**) Time course of fluctuation of the longitudinal position of the ID section in (**A**). The SD of fluctuation was 83 nm during the course of observation (0.32 s). The heart in an open-chest mouse was illuminated using a laser at 532 nm. Imaging was performed at 34 frames/s.

First, we confirmed that the myocardial membrane systems, including the intercellular junction, sarcolemma, and T-tubules, can be visualized by CellMask in the LV of the arrested mouse heart in vivo (Figure 2A, Movie S1) (as in References [24,25]). Given the nature of CellMask in that it enables fast and uniform labeling of the plasma membrane in virtually all living cells, it is reasonable to consider that the CellMask-stained ID in Figure 2A consists of the GJ, perinexii, adherens junctions, and desmosomes (see Figure 1A and Introduction).

We performed a fluctuation analysis for longitudinal movements of a section of the CellMask-stained ID ((see yellow rectangle in Figure 2A with a lateral width of 1 pixel (=210 nm)), and found that the standard deviation (SD) of fluctuation was 83 nm (Figure 2B). We previously reported that the SD of the length of a single SL in a myocyte in the *α*-actinin-AcGFP-expressing heart at rest was 20 nm in vivo [26]. Yet, the value was ~4 times larger, which is consistent with preferential longitudinal deformation of the ID and is distinct from that of other cellular structures.

**Figure 3:**
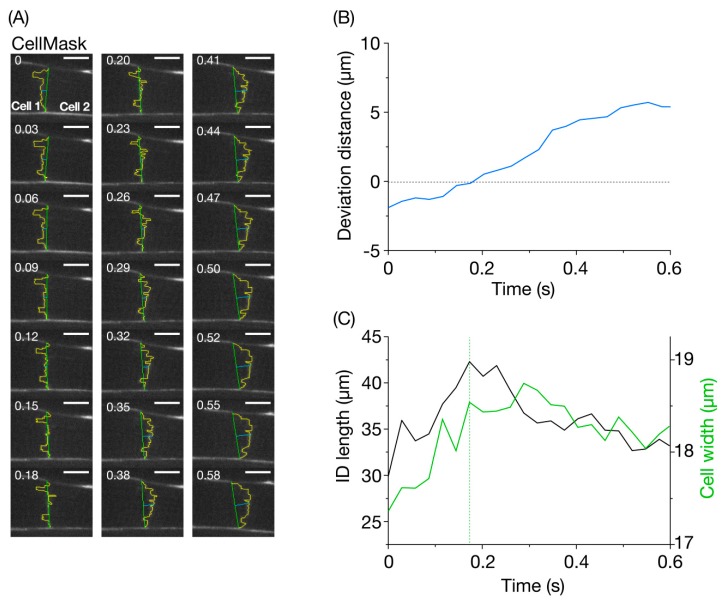
Imaging of the intercalated disc in the CellMask-treated left ventricle of the living mouse heart in vivo. (**A**) Confocal images of the intercalated disc (ID) at varying time points. Cell 1 and Cell 2 are located on the left and right side of the ID, respectively. Number on the top left in each figure indicates the time point at which the image was obtained. The ID was traced by a yellow line. Vertical green line, cell width, horizontal blue line, deviation of the ID at the center from the mid-point of vertical green line (see Materials and Methods). The heart in an open-chest mouse was excited using a laser at 532 nm, and imaging was performed at 34 frames/s for 0.58 s. Bar on top right, 10 μm. (**B**) Time course of changes in the deviation distance for the ID (shown in blue). Deviation distance was defined as the length of the horizontal blue line in (**A**). The value became zero at 0.18 s. (**C**) Time course of changes in the ID length and cell width. Black line, ID length (total length of the yellow line in (**A**)); green line, cell width. Cell width represents the width of Cell 2 and Cell 1, respectively, before and after 0.18 s (at which deviation distance became zero, see (**B**)).

We then analyzed whether or not the CellMask-stained ID structure (traced in yellow in Figure 3A) changes during propagation of contraction from cell to cell, by taking advantage of a mouse with a low heart rate under deep anesthesia (see Materials and Methods). It was observed that the ID contour fluctuated dramatically with dynamic changes in both longitudinal position and shape over time (Figure 3A, Movies S2 and S3). In the presented example, contraction was propagated from Cell 1 at the left to the rightward Cell 2, during the recording period. At time 0.00 s, the ID was located on the left side of the cell width line (shown in green), which forms a convex shape toward the leftward and contracting Cell 1. The ID contour then moved to the right, becoming largely superimposed on the cell width line at 0.15 and 0.18 s (i.e., deviation distance <0.5 μm). Thereafter, since contraction was initiated in Cell 2, the ID was deformed into a rightward convex shape. 

Figure 3B shows a time-dependent change in the ”deviation distance” (displacement of the center of the CellMask-stained ID and cell width line, cf. Figure 3A). The measured deviation changed during the propagation of contraction between cells with a nearly monotonic increase reaching a peak at 0.55 s. In this case, negative and positive values indicate the position of the mid-section of the ID on the side of Cell 1 and Cell 2, respectively (see Materials and Methods). In this case, the peak negative and positive values were −1.9 and 5.4 μm, respectively (cf. Movies S2 and S3). Thus, there was a large amplitude change in deviation distance of ~7.3 μm. 

We then analyzed the time-dependent change in the ID length as compared with that of the cell width line length (Figure 3C). Our analysis revealed that, during the propagating contraction in Figure 3A, the ID length changed by as much as ~12 μm, i.e., between ~30 and ~42 μm. This is followed by an increase in cell width measured at the junction between the two cells. The marked change in the ID length, which was as much as ~30% in this case, indicates flexibility upon propagation of contraction to a neighboring myocyte, i.e., from Cell 1 to Cell 2 (as indicated by Figure 3B). The peak of elongation of the ID length coincided with the time at which the deviation distance became ~zero (i.e., 0.18 s). Afterward, the ID length decreased as time progressed, and reached a value similar to that at the onset of imaging (i.e., ~33 μm at 0.55 s). Likewise, the cell width concomitantly decreased. Reportedly, during a twitch contraction in rat and ferret ventricular cells, there is an increase in cell width (~6–7%), but no significant change in the cell volume (i.e., constant volume) because the cells decrease in length (~12%) [28]. Although this previous study was performed on isolated cardiomyocytes, it is reasonable to consider that the same scenario applies to myocytes in the intact heart in vivo. We consider that, in the time course of a change in the widths of two adjoining cells, the ascending limb (cell width line in Cell 2) indicates the time during which Cell 2 is contracting while the descending limb (cell width line in Cell 1) is the time during which Cell 1 is relaxing and, hence, stretched by Cell 2. 

To the best of our knowledge, this is the first study demonstrating fluctuating movements of the ID in the living heart in vivo, which indicates that this structure can be deformed by as much as ~30% (Figure 3C). As reported previously [3], the GJ and perinexii are located in the plicate folded plasma membrane regions of the ID. Because stretching the plasma membranes by ~40% is likely to cause irreversible damage to their structure, it is likely that the elongation of the ID length results, at least in part, from the unfolding of the folded membrane regions for the GJ and perinexii. What is the mechanism by which flexible movements of the ID membrane occur during propagation of contraction from cell to cell? Within the ID, myofibrils from both sides of cells are intricated, which makes rugged membrane structures consist of the GJ and perinexii, adherens junctions, and desmosome (see Figure 1, cf. [6,12]). As previously demonstrated by us [26,27], sarcomeric motions are not synchronized even in the same myocyte when the heart is at work in vivo. Therefore, we consider that, within the same myocyte, the shortening and lengthening motions of individual myofibrils are not completely in synchrony, and any out-of-phase movements of myofibrils between connecting myocytes, albeit by a slight magnitude, may mechanically cause distortions of the ID structure. As the imaging in the present study was performed at a low heart rate to avoid an out-of-focus problem (i.e., to maximally enhance precision), future studies are needed to systematically investigate the following: (1) whether and where folding/unfolding repeatedly occurs in the folded membrane regions in the ID at various time points during the cardiac cycle, and (2) the relationship between heart rate and the magnitude of ID fluctuation occurs not only in normal but also in failing hearts under various conditions.

What is the physiological significance of flexible movements of the GJ and perinexii in the heart? It is likely that, when stretched in lateral directions, unfolding of plasma membranes occurs, which results in an increase of the lateral length of the ID. Mechanical load would be expected to be concomitantly transmitted two-dimensionally within the ID, including the GJ and perinexii. As described in the Introduction, the traditional role of Cx43 in the GJ in transmission of the electrical impulse to longitudinally connected cardiomyocytes (electronic conduction) is now a controversial topic. At the least, it is safe to state that electronic conduction is never the sole factor that accounts for effective propagation of the action potential in the heart. Accumulating evidence has demonstrated that Na_v_1.5, expressed in cardiomyocytes along with Cx43 in the perinexii (and presumably in the GJ), plays an important role in propagation of depolarization in the longitudinal direction (ephaptic conductance, see References [18,19,29]). In this case, it is important that Na_v_1.5 operates as a “mechano-sensitive” channel in that the kinetics of activation or inactivation are accelerated with stretch in a reversible manner [29,30]. It has been reported that the sodium current via Na_v_1.5 is increased by stretch in cardiomyocytes [31]. It is, therefore, possible that the mechanical load on Na_v_1.5 upon stretch of the perinexii increases its open probability, which gives rise to acceleration of propagation of depolarization. Consistent with this view, the present study demonstrated that contraction and relaxation occurred almost concomitantly with, respectively, elongation and shortening of the ID length (see Cell 2 and Cell 1 for contraction and relaxation behaviors, respectively, Figure 3C). Future studies are needed to systematically investigate the following: **(1)** how Na_v_1.5 current in the perinexii varies depending on the mechanical load in the ID, and **(2)** how these effects modulate action potential propagation between cells not only in normal hearts but also in failing hearts.

It is worthwhile to note that continual folding/unfolding of the ID membranes may sustain high expression levels of Cx43 in the GJ (and perinexii). Accumulating evidence shows that repeated stretching of the membranes enhances Cx43 expression in neonatal rat cardiomyocytes [32]. Therefore, it is safe to consider that this scenario applies to the adult heart in that an ongoing and intermittent load on the GJ (and perinexii) may sustain high expression levels of Cx43 in the GJ (and perinexii) by promoting electronic conduction. 

**Figure 4:**
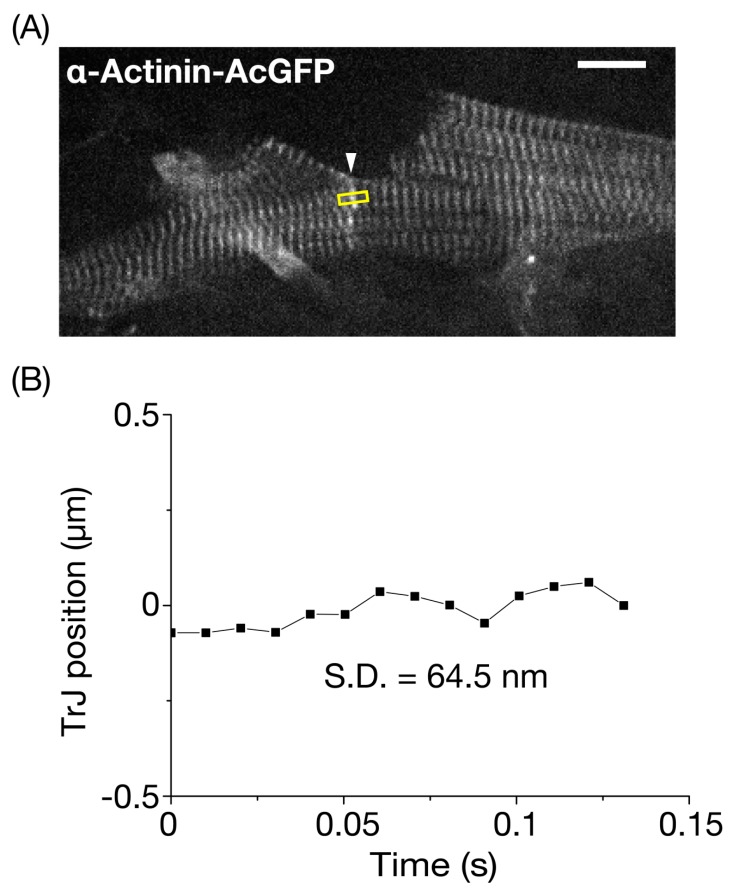
Precision analysis for the longitudinal position of the transitional junction in the resting mouse left ventricle. (**A**) In vivo confocal image of neighboring myocytes revealed transitional junctions (TrJ), which appeared as a singlet band with a high fluorescence signal (arrow, see also description in text). *α*-Actinin-AcGFP was expressed in myocytes in the surface of the left ventricle (LV) based on our previously reported procedure (see Materials and Methods). The longitudinal fluctuation of an TrJ section (width, 5 pixels (=1.05 μm)) in the yellow rectangle was analyzed under the arrested condition via deep anesthesia with >~5% isoflurane. Bar on the top right indicates 10 μm. (**B**) Time course of fluctuation of the longitudinal position of the TrJ section in (**A**). The SD of fluctuation was 64.5 nm during the course of observation (0.13 s). The heart in an open-chest mouse was excited using a laser at 488 nm. Imaging was performed at 99 frames/s.

Previously, we analyzed changes in the lengths of sequentially connecting sarcomeres in an *α*-actinin-AcGFP-expressing myocyte in the LV of the beating mouse heart in vivo [26,27]. In the present study, we analyzed movements of the intercellular junction visualized by *α*-actinin-AcGFP expression in the LV of a living mouse. An ADV injection enabled visualization of striations in two longitudinally connecting myocytes (Figure 4A, Movie S4), and the fluorescent image of the interconnecting region was noted to be thicker and fuzzier than that of normal Z discs. Bennett et al. [33] reported that, in the final half-sarcomere adjacent to the ID, there was a standard I-band that ended with a region that resembled a Z disc where α-actinin and N-terminal titin were expressed. They designated this region of the TrJ, and beyond the TrJ toward the membrane of the ID, the actin and associated proteins of the thin filaments in the ID folds were not sarcomeric. Likewise, more recent studies demonstrated that the TrJ has an immature Z disc-like structure [6]. These studies led us to conclude that the AcGFP fluorescent signal at the edge of a myocyte originates from the TrJ. It has been reported that the width of a single cardiac myofibril of adult mammals is ~1–2 μm [34]. Therefore, given that the lateral width of the TrJ was ~3 μm in Figure 4A, we consider that the TrJ extends over a few myofibrils at the edge of a myocyte. The SD of the longitudinal movement of a section of the TrJ (5 pixel) between myocytes of the arrested heart in vivo was 64.5 nm (Figure 4B).

**Figure 5:**
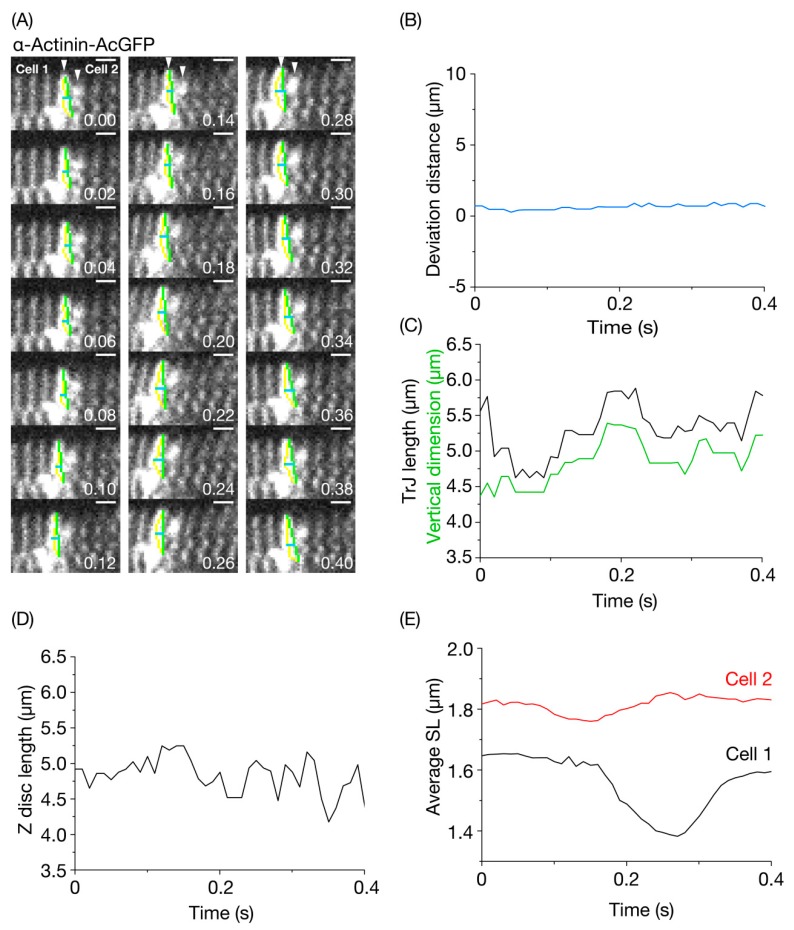
Imaging of the transitional junction in the *α*-actinin-AcGFP-expressing left ventricle of the living mouse heart in vivo. (**A**) Confocal images of the transitional junction (TrJ) at varying time points. Cell 1 and Cell 2 are located on the left and right side of the TrJ, respectively. Number on bottom right in each figure indicates the time point at which the image was obtained. The TrJ in Cell 1 is shown by a yellow trace. Green line, vertical dimension of the TrJ. Horizontal blue line, deviation of the central part of the TrJ from the mid-point of vertical green line (see Materials and Methods). The heart in an open-chest mouse was illuminated using a laser at 488 nm, and imaging was performed at 99 frames/s. Bar on top right, 2 μm. See Movies S5 and S6. (**B**) Time course of changes in the deviation distance for the TrJ (shown in blue). Deviation distance was defined as the length of the horizontal blue line in (**A**). Changes in the deviation distance was very small (~0.8 μm) as compared to Figure 2B (i.e., CellMask treatment). (**C**) Time course of changes in the TrJ length and vertical dimension. Black line, TrJ length (total length of yellow line in (**A**)); green line, vertical dimension. The TrJ length and vertical dimension changed closely. The magnitude of change in the TrJ was on average <~20%. (**D**) Time course of changes in the Z disc length. The Z disc located just next to the TrJ in Cell 1 was used for analysis. (**E**) Time-course of changes in mean SL in Cell 1 (black line) and Cell 2 (red line). The average length of 10 sarcomeres next to the TrJ was analyzed for both Cell 1 and Cell 2.

We then analyzed whether or not the TrJ structure is altered during propagation of contraction from cell to cell in the LV of a living mouse in vivo. The fluorescent signal of *α*-actinin-AcGFP in the TrJ between myocytes in the arrested heart that appeared was a singlet (Figure 4A), but, at a low heart rate (see Materials and Methods), it became a doublet in the heart at work (see arrows in Figure 5A) (Movies S5 and S6). Figure 5A shows two TrJ (one from Cell 1 (left) and the other from Cell 2 (right)) at various time points from zero to 0.40 s. Because the TrJ length in Cell 1 was longer than that in Cell 2 with a constant fluorescent signal during the course of imaging (coupled presumably with a lesser defocus problem), we analyzed the former in the present study. In contrast to the data with CellMask (cf. Figure 3A, Movies S2 and S3), the change of the TrJ structure was very small during the course of imaging. Compared to the data on the CellMask-stained ID whose deviation distance changed by as much as ~7.3 μm. Values for the TrJ was within ~0.5 μm (Figure 5B). 

Figure 5C shows time-dependent changes in the TrJ length and width of myofibrils measured at the position of the TrJ. The two parameters changed in parallel with time with an observed amplitude of deformation of ~1.3 μm for the TrJ (i.e., ~20%). However, no clear pattern was observed for the time-dependent change in the length of the Z disc adjacent to the measured TrJ (Figure 5D). These findings are consistent with the notion that, while the TrJ is easily distorted upon movements of individual myofibrils, due presumably to its immature structure [6], normal Z discs connected laterally by structural proteins, such as desmin [35,36,37,38] have a comparatively solid structure. Since the imaging in the present study was performed at a low heart rate (see above), future studies are needed to systematically investigate the relationship between heart rate and the magnitude of TrJ distortion not only in normal hearts but also in failing hearts under various conditions. 

We then analyzed the time-course of changes in SL in Cell 1 and Cell 2 (Figure 5E). During the course of imaging, Cell 2 started to shorten at 0.08 s, with the peak of shortening occurring at 0.15 s. This was followed by lengthening, which ceased at 0.26 s. Cell 1 started to shorten at 0.16 s (at which time, Cell 2 exhibited peak shortening) with the peak of shortening occurring at 0.27 s. This was followed by lengthening that ceased at 0.40 s. Therefore, during the observation period, contraction propagated from Cell 2 to Cell 1 (i.e., from right to left, in this case). Likewise, these SL analyses revealed that the TrJ width peaked during propagation of contraction from Cell 2 to Cell 1 (at ~0.2 s). This is similar to the result with CellMask in that the width of the cell-cell junction became maximal at the time when contraction was propagated (from Cell 1 to Cell 2 in Figure 3C).

The diastolic SL as well as the SL shortening ratio differed markedly in Cell 1 and Cell 2 (diastolic SL, 1.65 and 1.85 μm in Cell 1 and Cell 2, respectively. SL shortening ratio, ~16% and ~5% in Cell 1 and Cell 2, respectively). These findings suggest that the SL dynamic behaviors vary markedly between myocytes in vivo, but the electric impulse and the tug-of-war of force between myocytes along the conduction system organize rhythmic contractions of the heart. One may point out that the average SL was ~1.38 μm, i.e., shorter than the sarcomeric A-band length (~1.6 μm, [39]) at the peak of shortening in Cell 1. We consider that, under such conditions, the A-bands are distorted or buckled along myofibrils, and, therefore, SL (as indexed by the distance between the Z discs expressing *α*-actinin-AcGFP) becomes apparently shorter than the A-band length on the confocal plane.

In conclusion, we demonstrated in the in vivo heart of the mouse that the ID membrane had a markedly flexible structure in that its length was changed by as much as ~30% during propagation of contraction from cell to cell. Given the previous findings with electron microscopy that the plasma membranes within the ID are folded [3,4], the present findings suggest that folding/unfolding of the membrane repeats could accelerate ephaptic conduction via activation of Na_v_1.5, in coordination with Cx43-based electric conduction. Application of α-actinin-AcGFP expression by ADV in the LV of the mouse heart enabled visualization of the TrJ whose lateral length changed during propagation of contraction from cell to cell by ~20%, which indicates that structural distortion is likely to occur in this region.

## Supplementary Materials

The following are available online at https://www.mdpi.com/2079-4991/10/3/532/s1, Movie S1: Real-time tracking of intercalated disc positioning in the resting mouse left ventricle. CellMask-labeling was employed to image cardiomyocyte membranes, including the intercalated disk, in the central region of the left ventricle (see Figure 2A). The heart was arrested by deep anesthesia with >~5% isoflurane. Objective lens, 60× (N/A, 1.00; water immersion); acquisition cycle, 34.38 frames/s; exposure time, 0.02882 s. Bar, 10 μm, Movie S2: Movement of the intercalated disc in the contracting mouse left ventricle. The intercalated disc is apparent between two contracting CellMask-stained myocytes in the left ventricle epicardium (cf. Figure 3A). The image sequence was obtained under deep anesthesia with ~5% isoflurane. Objective lens, 60× (N/A, 1.00; water immersion); acquisition cycle, 34.38 frames/s; exposure time, 0.02882 s. Bar, 20 μm, Movie S3: Movement of the intercalated disc during contraction with superimposed tracking. The recording in Movie S2 is presented with key features traced, including the intercalated disc (yellow), cell width (green), and deviation distance (blue). Objective lens, 60× (N/A, 1.00; water immersion); acquisition cycle, 34.38 frames/s; exposure time, 0.02882 s. Bar, 10 μm, Movie S4: Real-time tracking of transitional junctions in the resting mouse left ventricle. α-Actinin-AcGFP-expressing myocytes were used to image transitional junctions in the central region of the mouse left ventricle (see Figure 4A). The heart was arrested by deep anesthesia with >~5% isoflurane. Objective lens, 60× (N/A, 1.00; water immersion); acquisition cycle, 99.21 frames/s; exposure time, 0.00982 s. Bar, 10 μm, Movie S5: Movements of transitional junctions in the contracting mouse left ventricle. Transitional junctions are apparent between two α-actinin-AcGFP-expressing myocytes in the left ventricular epicardium (see white arrow at 0.00 s; cf. Figure 5A). The image sequence was obtained under deep anesthesia with ~5% isoflurane. Objective lens, 60× (N/A, 1.00; water immersion); acquisition cycle, 99.206 frames/s; exposure time, 0.00982 s. Bar, 20 μm, Movie S6: Movements of transitional junctions during contraction with superimposed tracking. The recording in Movie S5 is presented with key features traced, including the transitional junction (yellow), vertical dimension (green) and deviation distance (blue) in Cell 1 (see Figure 5A). Objective lens, 60× (N/A, 1.00; water immersion); acquisition cycle, 99.206 frames/s; exposure time, 0.00982 s. Bar, 2 μm.

## Abbreviations

ADV, adenovirus vector. [Ca^2+^]*_i_,* intracellular Ca^2+^ concentration. Cx43, connexin 43. ECG, electrocardiogram. F.I., fluorescence intensity. GJ, gap junction. ID, intercalated disc. LV, left ventricle. SD, standard deviation. SL, sarcomere length. SR, sarcoplasmic reticulum. TrJ, transitional junction.
