# Supplementary material for: Real-Time In Vivo Imaging of Mouse Left Ventricle Reveals Fluctuating Movements of the Intercalated Discs
Source: Nanomaterials (Basel). 2020 Mar 16;10(3):532. doi: 10.3390/nano10030532 (PMC7153594; doi:10.3390/nano10030532)
Supplement: Supplementary file 1 [file nanomaterials-10-00532-s001.zip › Supplementary Movies/Suplementary movie legends.docx]

**Supplementary movie legends**

**Movie S1: Real-time tracking of intercalated disc positioning in the resting mouse left ventricle.** CellMask-labeling was employed to image cardiomyocyte membranes, including the intercalated disk, in the central region of the left ventricle (see **Figure 2A**). The heart was arrested by deep anesthesia with >~5% isoflurane. Objective lens, 60× (N/A, 1.00; water immersion); acquisition cycle, 34.38 frames/s; exposure time, 0.02882 s. Bar, 10 μm.

**Movie S2: Movement of the intercalated disc in the contracting mouse left ventricle.** The intercalated disc is apparent between two contracting CellMask-stained myocytes in the left ventricle epicardium (cf. **Figure 3A**). The image sequence was obtained under deep anesthesia with ~5% isoflurane. Objective lens, 60× (N/A, 1.00; water immersion); acquisition cycle, 34.38 frames/s; exposure time, 0.02882 s. Bar, 20 μm.

**Movie S3: Movement of the intercalated disc during contraction with superimposed tracking.** The recording in **Movie S2** is presented with key features traced, including the intercalated disc (yellow), cell width (green), and deviation distance (blue). Objective lens, 60× (N/A, 1.00; water immersion); acquisition cycle, 34.38 frames/s; exposure time, 0.02882 s. Bar, 10 μm.

**Movie S4: Real-time tracking of transitional junctions in the resting mouse left ventricle.** α-Actinin-AcGFP-expressing myocytes were used to image transitional junctions in the central region of the mouse left ventricle (see **Figure 4A**). The heart was arrested by deep anesthesia with >~5% isoflurane. Objective lens, 60× (N/A, 1.00; water immersion); acquisition cycle, 99.21 frames/s; exposure time, 0.00982 s. Bar, 10 μm.

**Movie S5: Movements of transitional junctions in the contracting mouse left ventricle.** Transitional junctions are apparent between two α-actinin-AcGFP-expressing myocytes in the left ventricular epicardium (see white arrow at 0.00 s; cf. **Figure 5A**). The image sequence was obtained under deep anesthesia with ~5% isoflurane. Objective lens, 60× (N/A, 1.00; water immersion); acquisition cycle, 99.206 frames/s; exposure time, 0.00982 s. Bar, 20 μm.

**Movie S6: Movements of transitional junctions during contraction with superimposed tracking.** The recording in **Movie S5** is presented with key features traced, including the transitional junction (yellow), vertical dimension (green) and deviation distance (blue) in Cell 1 (see **Figure 5A**). Objective lens, 60× (N/A, 1.00; water immersion); acquisition cycle, 99.206 frames/s; exposure time, 0.00982 s. Bar, 2 μm.
